# Supplementary material for: Fussing About Fission: Defining Variety Among Mainstream and Exotic Apicomplexan Cell Division Modes
Source: Front Cell Infect Microbiol. 2020 Jun 5;10:269. doi: 10.3389/fcimb.2020.00269 (PMC7289922; doi:10.3389/fcimb.2020.00269)
Supplement: Supplementary file 1 [file Data_Sheet_1.docx]

**Supplementary Material**

**Figure S1**


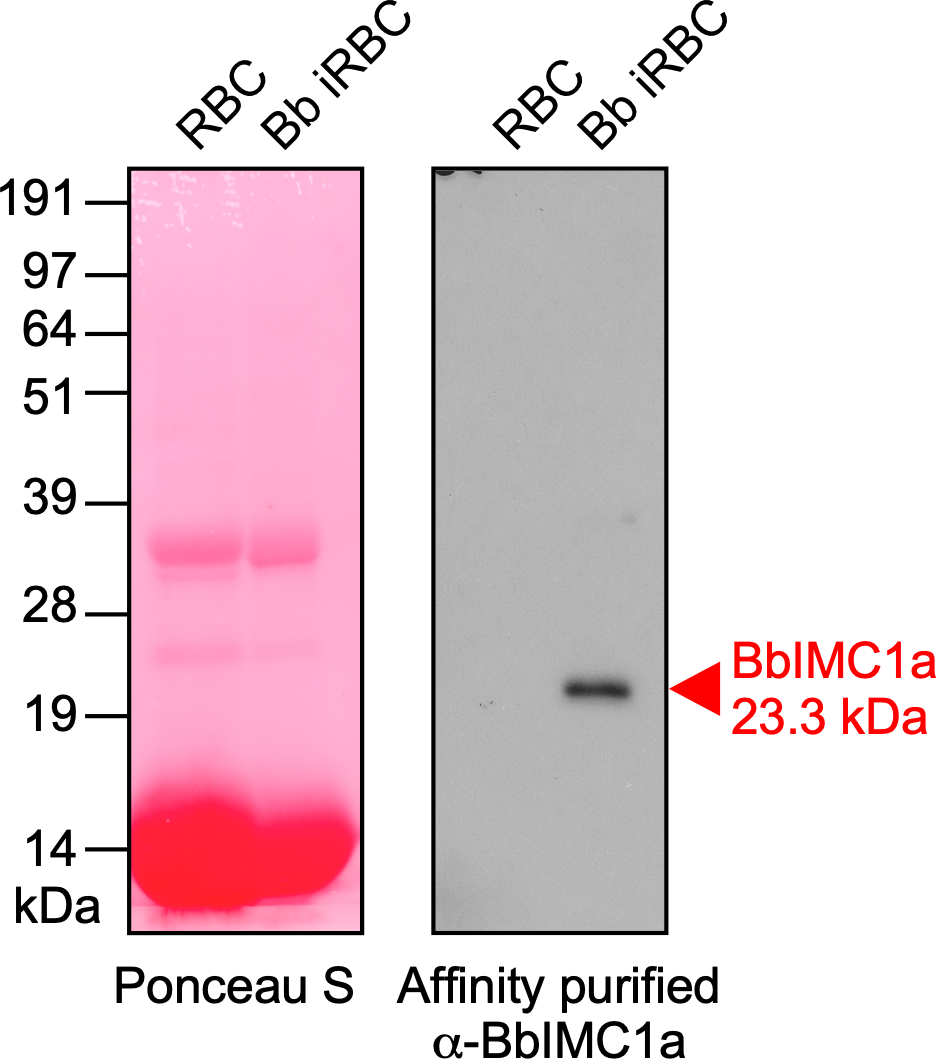


**Figure S1. Validation of affinity purified guinea pig antiserum raised against recombinant His6-BbIMC1a by western blot.** Left panel: PonceauS staining of the western blot serving as loading control; Right panel: serum affinity purified against recombinant His6-BbIMC1a diluted 1:250. RBC indicates cow red blood cell total lysate; Bb iRBC indicates total lysate of cow red blood cells with a *B. bigemina* parasitemia of 12%. Equal amounts of lysate were loaded across lanes. The predicted MW of BbIMC1a is 23.3 kDa.
